# The Neural Basis of Moral Judgement for Self and for Others: Evidence From Event-Related Potentials

**DOI:** 10.3389/fnhum.2022.919499

**Published:** 2022-05-27

**Authors:** Qin Jiang, Linglin Zhuo, Qi Wang, Wenxia Lin

**Affiliations:** ^1^Research Center of Psychology and Education, School of Marxism, Guangxi University, Nanning, China; ^2^Department of Psychology, Sun Yat-sen University, Guangzhou, China

**Keywords:** moral judgment, theory of mind, self, others, ERP

## Abstract

Developmental and neuroscience works have demonstrated that the moral judgment is influenced by theory of mind (ToM), which refers to the ability to represent the mental states of different agents. However, the neural and cognitive time course of interactions between moral judgment and ToM remains unclear. The present event-related potential (ERP) study investigated the underlying neural substrate of the interaction between moral judgment and ToM by contrasting the ERPs elicited by moral judgments for self and for others in moral dilemmas. In classic moral dilemmas, the agents must choose between the utilitarian choice (taking the action to kill or harm an innocent person but saving more people) and the non-utilitarian choice (taking no action to kill or harm the innocent person but letting some people die). The ERPs were recorded from participants who made moral judgments for self and for others when the agent made utilitarian or non-utilitarian choices during the dilemma. The results revealed that the moral judgment for others elicited a larger frontal late positive component (LPC, 500–900 ms) than that for self when the agents made utilitarian choices, while no difference was observed on early components of N1, P2, and N2. Moreover, individual differences in mentalizing ability were negatively correlated with the LPC amplitudes. These findings suggested that ToM modulated the late controlled process but not the early automatic process during moral judgments.

## Introduction

In everyday lives, people usually tend to make a moral judgment by evaluating the appropriateness or moral permissibility of the agent’s actions in a moral dilemma. However, people do not always make a moral judgment fairly and objectively. Moral hypocrisy is a very common bias in moral judgment. It refers to that individuals make more severe moral judgment when a moral transgression is enacted by others than an identical transgression enacted by themselves (Valdesolo and DeSteno, 2008). This bias has been shown to extend across members of a group even across tribes. For example, a moral transgression enacted by an ingroup member seems more acceptable than the identical behavior enacted by an outsider (Valdesolo and DeSteno, 2007).

Theory of mind (ToM), the capacity to represent the mental states of different agents, is considered as a fundamental component of moral judgment (Blair et al., 2006; Young and Saxe, 2008; Young and Dungan, 2012; Decety and Cowell, 2014). Typically, the moral judgment of an action not only depends on its consequences but also the mental states of the agent at the time of the action. As in the scenario used by Young and Saxe (2008, 2009a,b) in their studies, a daycare worker served spoiled meat to the children in his care. When people made a moral judgment of the behavior, they considered the worker’s beliefs or intentions. For instance, whether the worker believed that the meat was fresh because of its sealed package and expiration date, or he knew the meat was spoiled, but still cooked it with the intention to save money? Besides, the desires and emotions of children might also influence the moral judgment of the worker’s action. Depending on the diverse mental states, people would regard the same action as morally blameworthy or forgivable.

The association between moral judgment and ToM have been verified by developmental studies. For example, 4-year-old children’ justifications concerning their views on the permissibility of transgressions involving friends were correlated with understanding of beliefs and emotions (Dunn et al., 2000). Another developmental study by Baird and Astington (2004) found that children could utilize information about people’s intentions to make moral distinctions between identical actions. Furthermore, this ability was positively related to children’s understanding of false belief. Two recent studies showed that children’s ToM competence was predictive of personal moral judgments and varied based on the ingroup/outgroup status or the intention of the target (D’Esterre et al., 2018; Glidden et al., 2021).

Neuroimaging studies on the neural correlates of moral processing showed that several brain areas were involved in moral judgments including ventromedial prefrontal cortex, dorsolateral prefrontal cortex, medial prefrontal cortex (MPFC), temporo-parietal junction (TPJ), superior temporal sulcus, precuneus, amygdala, and insula (Moll et al., 2002a,b; Young and Saxe, 2008, 2009a,b; Young and Dungan, 2012; Decety and Cowell, 2014). These regions have also been suggested to be the underpinning of ToM processing (Mitchell et al., 2005; Amodio and Frith, 2006; Sommer et al., 2007; Saxe, 2010; Young et al., 2010b; Mahy et al., 2014). For example, Young and Saxe (2008) observed increased activity in the right TPJ, precuneus, and MPFC when participants are making moral judgments, which might suggest that moral judgments elicited spontaneous mental state inference. Young et al. (2010a) used transcranial magnetic stimulation to disrupt neural activity in the right TPJ transiently before and during moral judgment. The results revealed that interfering with activity in the right TPJ disrupted the capacity to use mental states in moral judgment. It made participants tend to judge attempted harms as less morally forbidden and more morally permissible.

Since the evidence from both developmental and neuroscience works demonstrated the vital role of ToM in moral judgment, the aforementioned moral bias is likely to be interpreted on the basis of ToM. For instance, adopting the perspective of the outgroup members, or understanding their mental states, leads to a decrease of the stereotypes for the individuals and more positive evaluations of the whole group (Decety and Cowell, 2014). On the other hand, understanding the mental states of the ingroup members, which usually be much easier, can interfere with moral judgment by introducing partiality for ingroup members. Furthermore, compared to taking the perspectives of another, people are more efficient when making judgments from their own perspectives (Wang et al., 2015). The activation of self-perspective seems spontaneous and automatic in both children and adults (Royzman et al., 2003; Birch and Bloom, 2004; Apperly et al., 2009). These findings indicate that the relations between ToM and moral judgment may be modulated by the interpersonal variables. However, the neural and cognitive time courses of interactions between ToM and moral judgment remains unclear. In terms of the high temporal resolution offered, the event-related potential (ERP) methodology provides an opportunity for researchers to explore these issues.

The moral tasks used in ERP studies concerning moral processing can be grouped into two categories: moral decision tasks and moral judgment tasks (Garrigan et al., 2016). For example, participants were asked to answer “Would you do X?” in moral decision tasks and “Is it acceptable to do X?” in moral judgment tasks. Discrepancy has been found between the two categories of tasks in both brain activation patterns and behavior responses (Tassy et al., 2013; Garrigan et al., 2016). Previous ERP studies found that the interpersonal relationship not only modulates the behavioral performance but also the neural responses during moral decision-making (Chen et al., 2009; Zhan et al., 2018). In work by Chen et al. (2009), people were required to decide who to rescue when two relatives (e.g., father and mother) or two strangers (e.g., stranger A and stranger B) were buried in the debris after an earthquake. Results showed that people took longer time to make decisions pertaining to relatives than strangers. ERP analysis revealed that making moral decisions for relatives elicited a greater P2 (190–210 ms) and P3 (350–450 ms) than for strangers. The authors proposed that more attentional resources and stronger cognitive conflict were involved in moral decision-making for relatives than for strangers. Another study by Zhan et al. (2018) compared the neural and behavioral responses during moral decision-making under different intimate interpersonal relationships (friend, acquaintance, or stranger). Results showed that participants made more egoistical decisions with longer reaction times and experienced more unpleasure emotion for strangers versus friends and acquaintances. Furthermore, the moral decision-making for strangers elicited larger P260 and late positive potentials (LPP, 300–450 ms) than that for friends and acquaintances, whilst these differences were not observed between the latter two groups. The authors argued that the P260 reflects the dilemma conflicts and negative emotional responses, while the LPP indexes the cognitive appraisal and reasoning processes. The results suggested that the moral decision-making for people close to oneself elicited the weaker conflicts and emotional responses, and required fewer attentional resources or cognitive effort to deal with the conflicts.

The larger P260 and slow waves (450–600 ms) were also observed when participants made decisions in the instrumental moral dilemmas (i.e., self-involvement) rather than the incidental moral dilemmas (i.e., non-self-involvement) (Sarlo et al., 2012). In another ERP study by Sarlo et al. (2014), the trait empathy of the participants was measured by the scale of the Interpersonal Reactivity Index (IRI). The results found that the personal feelings of anxiety and discomfort in response to others’ distress (the score of Personal Distress subscale of the IRI) could predict the mean amplitudes of the P260 positively in Footbridge-type moral dilemmas. This finding suggested that the capacity of sharing feelings when witnessing another’s negative experience could mediate the emotion-related cortical activity in moral decision-making.

In these studies, participants were presented a hypothetical moral dilemma, and asked to make decisions about what they would do in the scenarios. These studies revealed the neural and cognitive time course of the interpersonal relationship impacts on moral decision-making; however, it remains unclear how and when ToM influences moral judgments. Besides, existing ERP studies focused on the moral processing for other persons with different interpersonal closeness to oneself, such as relatives versus strangers, or friends versus strangers. The moral judgments for self and for others have not been compared directly. As noted above, individuals usually make more severe moral judgment for others than themselves (Valdesolo and DeSteno, 2008). Exploring the neural and cognitive time course of moral judgments for self and for others can shed light on the interaction between ToM and moral judgment, and provide electrophysiological evidence for the interpretation of the common moral bias based on ToM. Thus, the present study aims to investigate the underlying neural substrate of the interaction between ToM and moral judgment by contrasting the ERPs elicited by moral judgments for self and for others.

Furthermore, the behavior manners of the protagonist in moral dilemmas should also be considered. In a classic moral dilemma, the protagonist usually has two options: taking the action to kill or harm an innocent person but saving more people or taking no action to the innocent person but letting some people die. Based on the Doctrine of Doing and Allowing (DDA) in moral processing, the harm caused by action is worse than the harm caused by omission (Cushman et al., 2006; Woollard, 2013). Taking the action of killing or harming one person to save more lives is considered as a type of utilitarian choice (maximizing benefits and minimizing costs), which elicited greater activation in brain areas involved in executive functions than the non-utilitarian choice (Greene et al., 2004). It was proposed that, compared to the non-utilitarian choice, the utilitarian choice triggered stronger emotional responses and required greater cognitive control to resolve the conflict between the negative emotions toward causing direct harm and rational computation (Greene et al., 2004; Sarlo et al., 2012, 2014).

In the present study, participants were asked to make moral judgments for self and for others when the agent chose to take the action or take no action during the dilemma. By contrasting the electrical activity elicited by moral judgments for different agents, the present study aimed to investigate the electrophysiological correlates underlying the interaction of ToM with moral judgments. Additionally, ToM was assessed using the “Reading the Mind in the Eyes” Test (RMET) (Baron-Cohen et al., 2001). The RMET was an “advanced theory of mind test” suitable for adult population. It was conceived as a measure of how well the participant can put themselves into the mind of the other person, and “tune in” to their mental state (Baron-Cohen et al., 2001), which was also referred to “mentalizing” (Frith et al., 1991). Furthermore, the present study can also provide neural evidence for the impact of DDA in moral processing by comparing the neural correlates underlying moral judgments for the choices of taking actions or just omission in moral dilemmas. According to ERP studies concerning ToM reasoning for different agents, the divergence of ERP waveforms between mental states reasoning for self and for others was observed on the later components such as the late positive component (LPC) and the late slow wave (LSW) (Jiang et al., 2016, 2021). Thus, we expect that the ERP components associated with interaction between ToM and moral judgment might appear in the later time window. Moreover, with respect to the DDA in moral processing, we expect that the ERP waveforms will be differentiated between moral judgments for the choices of taking the action and taking no action during the dilemma.

## Materials and Methods

### Participants

*A priori* power analysis was performed using the G*Power 3.1.9.7 (Faul et al., 2007). The result suggested that a minimum of 15 participants were required to reach the effect size f of 0.25 and the α error probability of 0.05 and the power (1-β error probability) of 0.95 in a repeated-measures analysis of variance (ANOVA) when the within-subjects experiment design was used in the present study.

To account for possible dropouts or errors during the test, twenty healthy adults were recruited to take part in this experiment. All were right-handed and with normal or corrected to normal vision. Three participants were excluded from the final sample because of excessive electrophysiological artifact (two participants) or because the participant felt uncomfortable and decided to quit (one participant). The remaining seventeen participants, including ten men and seven women, were aged between 18 and 23 years (*M* = 20.84 years, *SD* = 1.47 years). All participants signed an informed consent for the experiment before the testing and received monetary compensation for their participation. The experiment was approved by the local Academic Ethics Committee and was in accordance with the ethical guidelines of the Declaration of Helsinki.

### Stimuli and Task

The present moral dilemma task was based on a behavioral study by Lotto et al. (2013). Thirty moral dilemma scenarios were modified according to ERP technical demands and the experiment design. Each scenario described that the protagonist faced a dilemma situation in which a group of people were in urgent danger. The protagonist could take the action to save these people by killing or harming another innocent person. For example, four people were followed by a lion and the protagonist could save them by pushing a person off the tower to draw the lion toward him. If the protagonist took no action, then the four people would be caught by the lion.

A 2 (agent types: self or others) × 2 (behavior types: action or inaction) within-subjects design required all participants to complete four experimental conditions, including self-action, self-inaction, others-action, and others-inaction conditions. The structure of the four conditions was similar. Participants were asked to judge whether the behaviors of the agents in these moral dilemmas were appropriate.

### Procedure

For the self-action condition, in each trial, a moral dilemma was presented textually with the subject word “you”, which defined the agent of the behavior as being the participant themself. In this condition, the agent (a participants) took action to save the group of people in danger by killing or harming another innocent person. There was no time limit imposed on this screen of dilemma. Participants could press the “space” key to go to the next screen until they understood the dilemma. After a blank screen, participants were presented with the question “Is it appropriate for you to do so?” for 1,500 ms. The presentation of the question was the target event for ERP analysis. Next, two pictures were presented lasting for 250 ms as the answer options to the question. One picture was an upright thumb, representing a behavior deemed appropriate and approved. The other was an inverted thumb, representing the opposite. Participants provided their answers by pressing “F” for the left picture and “J” for the right picture. The positions of the two pictures were reversed in half of the trials.

For the self-inaction condition, the procedure was identical to that used in the self-action condition, with the exception that the agent (participant themself) chose to take no action of killing or harming another innocent person and the group of people’ lives were in danger.

For the others-action condition, the procedure was identical to that used in the self-action condition except that the protagonist of the dilemma was a person named “Zhangsan (张三)”, which defined the agent of the behaviors as other people. In Chinese culture, the “Zhangsan” name was usually referred to a normal adult male. It was chosen because most scenarios contain the actions requiring male physical strength. The target question “Is it appropriate for him to do so?” was adopted accordingly.

For the others-inaction condition, the procedure was identical to that used in the others-action condition, except that agent (Zhangsan, other people) took no action to the innocent person and the group of people’ lives were in danger.

**FIGURE 1:**
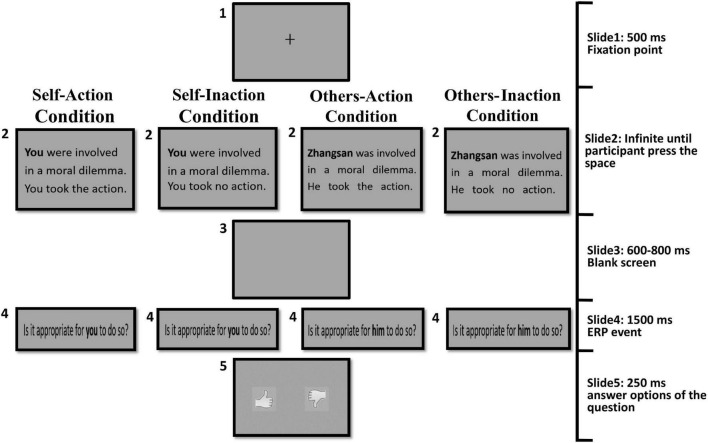
The procedure for the four experimental conditions.

Figure 1 illustrates the procedure for the four experimental conditions. There were 60 trials for each condition in the formal experiment (30 dilemmas were presented repeatedly twice). The total 240 experimental trials were randomly presented in four blocks. To ensure that the participants stay focused throughout the experiment, each block contained another four control trials in which participants responded to questions about some details of the dilemma scenarios. For example, “Were the four people followed by a lion?” or “Were the four people followed by a tiger?” Participants answered the questions in control trials by pressing “F” for the left option and “J” for the right option corresponding to “Yes” or “No”. The control trials were randomly interspersed among the experimental trials and were not included in the ERP analysis. Before the formal experiment, participants were asked to undertake sufficient training until they could fully understand the structure of the task. The dilemma scenarios used in training phase would not be presented in the formal experiment.

After the Electroencephalogram (EEG) recording session, participants completed the RMET (Baron-Cohen et al., 2001). In this test, participants were presented with a series of 36 photographs of the eye-region of human faces and were asked to choose a word best describing the feelings expressed by the person in the photograph from four options. The scores of the RMET ranged from 0 to 36, and the higher the score, the more mentalizing ability a person possessed.

### Behavioral Data Analysis

Reaction time (RT) was defined as the time between the test question onset and the key press. The proportion of approving was computed for each participant by dividing the number of the “thumbs-up” choice by the total number of response choice for each condition (n = 60). The preliminary inspection revealed that, the data of RT and the proportion of approving were normally distributed according to the Kolmogorov–Smirnov tests (*p*s > 0.074). Thus, a 2 (agent types: self or others) × 2 (behavior types: action or inaction) repeated-measures ANOVA was conducted for the RT data and the proportion of approving choices.

### Electrophysiological Recording and Analysis

Electroencephalogram was recorded from BrainAmp amplifiers (Brain Product, Herrsching, Germany) with 64 Ag/AgCl electrodes mounted on an elastic cap according to the International 10–20 system. The EEG signals were amplified by using a sampling rate of 500 Hz and a band pass of 0.016–100 Hz. Electrode impedances were maintained below 5 kΩ. Vertical electrooculogram (EOG) was monitored by an electrode below the right eye and horizontal EOG was monitored by an electrode at the external outer canthi of the left eye. The reference electrode was positioned at FCz and the ground electrode was positioned at AFz. In off-line analysis, all electrodes were re-referenced to average mastoids. A 0.016–30 Hz digital band pass filter and a 50 Hz notch filter were applied. EOG artifacts (eye blinks and movements) were excluded using an independent component analysis (ICA) algorithm. The segment epoch for ERPs was 1,200 ms, including the 200 ms pre-stimulus baseline and the 1,000 ms post-stimulus activity. Segments with an incorrect response or a peak-to-peak deflection exceeding ±100 μV were excluded from the final averaging. After artifact rejection, the average number of trials per condition submitted for final analysis was 58.7 for the self-action, 58.8 for the self-inaction, 59.0 for the others-action, and 58.8 for the others-inaction conditions, respectively.

**FIGURE 2:**
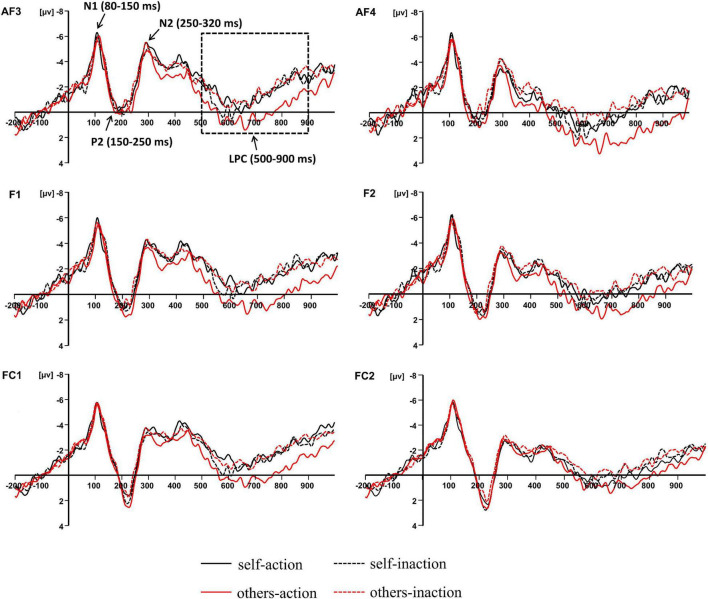
Grand-averaged event-related potential waveforms elicited by four experimental conditions at frontal electrode sites.

**FIGURE 3:**
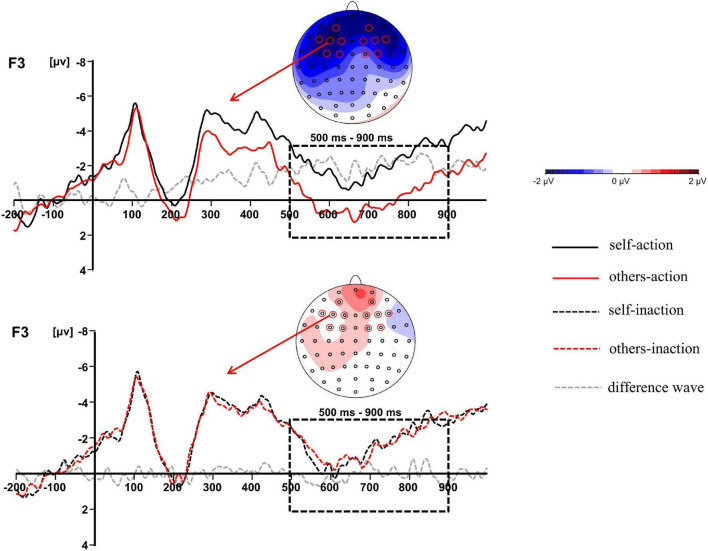
The difference waves at F3 and topographic maps of difference waves for LPC (500–900 ms): other-action condition subtracted from self-action condition (**top panel**), and other-inaction condition subtracted from self-inaction condition (**bottom panel**).

Previous studies concerning the neural bases of moral processing showed that moral judgment is associated with ERP components over the frontal region (Lahat et al., 2013; Sarlo et al., 2014; Gui et al., 2016; Decety and Cowell, 2017; Pletti et al., 2019). Visual inspection of the averaged waveforms of each condition and the topographic difference maps in the present study also suggested that the divergence of waveforms elicited by the four conditions distributed around frontal area (see Figures 2, 3). For these reasons, six pairs of frontal electrodes including AF3/AF4, F1/F2, F3/F4, F5/F6, FC1/FC2, and FC3/FC4, were selected for subsequent statistical analysis. As shown in Figure 2, four ERP components were elicited by time-locked stimulus including N1 (80–150 ms), P2 (150–250 ms), N2 (250–320 ms), and LPC (500–900 ms). The mean amplitudes of the above components were subjected to 6 × 2 × 2 × 2 repeated-measures ANOVA with electrode pairs (AF3/AF4, F1/F2, F3/F4, F5/F6, FC1/FC2, and FC3/FC4), hemisphere (left vs. right), agent types (self vs. others), and behavior types (action vs. inaction) as within-subjects variables. Statistical differences were reported as significant at *p* < 0.05. The Greenhouse-Geisser correction for non-sphericity was applied whenever appropriate. Bonferroni’s method was applied in both *post hoc* comparisons and simple effects analyses.

Lastly, to better understand the functional means of observed ERP effects, Pearson’s correlations were performed to investigate the association between neural and behavioral responses. The correlation between the RMET scores and the neural responses was also calculated.

## Results

### Behavioral Results

**FIGURE 4:**
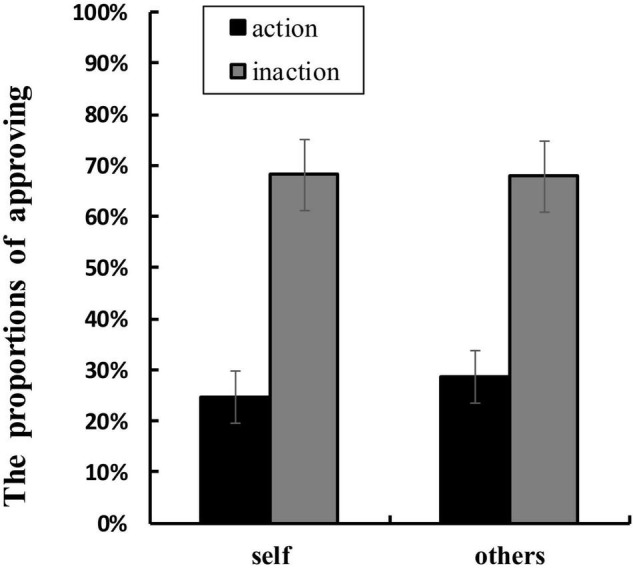
Mean proportions of approving (%) in each condition. The error bars show ± 1 standard error.

Table 1 shows the mean RTs and the proportions of approving in each condition. ANOVA of RTs showed that no main effect or interaction was significant. ANOVA of the proportions of approving found a significant main effect of agent types, *F*(1,16) = 4.707, *p* = 0.045, η_p_^2^ = 0.227. The proportion of approving for others conditions (*M* = 48.2%, *SD* = 0.140) was higher than that for self conditions (*M* = 46.5%, *SD* = 0.140). The main effect of behavior types was also significant, *F*(1,16) = 15.515, *p* = 0.001, η_p_^2^ = 0.492. The proportion of approving for inaction conditions (*M* = 68.0%, *SD* = 0.293) was greater than that for action conditions (*M* = 26.7%, *SD* = 0.219). Moreover, a significant interaction between agent types and behavior types was found, *F*(1,16) = 5.337, *p* = 0.035, η_*p*_^2^ = 0.250 (see Figure 4). Simple effects analyses showed that the proportion of approving in others-action conditions was greater than that in self-action conditions, *F*(1,16) = 18.049, *p* = 0.001, η*_p_*^2^ = 0.530, whilst the difference between the proportion of approving in others-inaction conditions and that in self-inaction conditions was not significant.

**TABLE 1:** Mean RTs (ms) and the proportions of approving (%) (standard deviation) in each condition.

	Self	Others
**RTs**		
Action	722.994 (169.134)	723.023 (174.017)
Inaction	717.392 (187.251)	700.407 (150.768)
**Proportions of approving**		
Action	24.8% (21.1)	28.5% (22.4)
Inaction	68.1% (29.6)	67.8% (28.8)

### Event-Related Potential Results

#### N1 (80–150 ms), P2 (150–250 ms), and N2 (250–320 ms)

There was no significant experimental main effect or interaction for the N1, P2, or N2 amplitudes.

#### LPC (500–900 ms)

The main effect of behavior types on the LPC amplitude was significant, *F*(1,16) = 14.011, *p* = 0.002, η_*p*_^2^ = 0.467. Action conditions (*M* = 0.205, *SD* = 4.366) elicited a more positive waveform than inaction conditions (*M* = −0.539, *SD* = 4.457). Moreover, there was a significant interaction between agent types and behavior types, *F*(1,16) = 5.218, *p* = 0.036, η_*p*_^2^ = 0.246. Simple effects analyses revealed that the moral judgment for others (*M* = 0.924, *SD* = 4.680) elicited a more positive waveform than that for self (*M* = −0.514, *SD* = 4.358) when the agents took actions to save a group of people by killing or harming another innocent person, *F*(1,16) = 6.277, *p* = 0.023, η_*p*_^2^ = 0.282, whilst the difference was not significant when the agents took no action. The results have been further confirmed by the topographic difference maps shown in Figure 3.

### Correlational Analyses

The amplitudes of the LPC were calculated by averaging the amplitudes from six pairs of frontal electrodes (AF3/AF4, F1/F2, F3/F4, F5/F6, FC1/FC2, and FC3/FC4). The results showed that, no correlation was found between the RTs and the LPC amplitudes, *r* = 0.270–0.437, *ps* > 0.080. The correlations between the proportions of approving choices and the LPC amplitudes were not significant, *r* = −0.002 to 0.223, *ps* > 0.389.

Participants’ RMET scores ranged from 16 to 28 (*M* = 21.65, *SD* = 2.644). Significant negative correlations were found between the RMET scores and the LPC amplitudes under each of the four experimental conditions (self-action: *r* = −0.499, *p* = 0.042, self-inaction: *r* = −0.564, *p* = 0.018, others-action: *r* = −0.490, *p* = 0.046, and others-inaction: *r* = 0.500, *p* = 0.041, respectively).

## Discussion

In the present study, we investigated the neural correlates underlying the interaction between ToM and moral judgment by contrasting the ERPs elicited by moral judgments for self and for others when the agent chose to take the action or take no action during the dilemma. The behavioral results revealed that the approval rating in the others-action condition was higher than that in self-action condition. The results suggested that participants perceived killing or harming an innocent person to save more people as more unacceptable when the action was taken by themselves than by other people. Interestingly, this finding did not support the moral hypocrisy in which individuals make more severe moral judgments for others than for themselves. On the contrary, it was consistent with what had been observed by Sarlo et al. (2012). Instrumental dilemmas (self-involvement) elicited a largely lower proportion of utilitarian choices than incidental dilemmas (no self-involvement). As noted above, utilitarian choice refers to choosing to kill or harm one person to save more lives in moral dilemmas. People were more reluctant to kill or harm an innocent person to save more when they themselves were involved in the scenarios. This possibly reflected that people were trying to avoid or constrain the stronger negative emotions toward personally killing or harming other innocent people. The behavioral finding was corresponding with the ERP results of LPC in the present study, which also revealed a significant interaction between the agent types and the behavior types, confirming the difference between the moral judgment for self and for others when the agent chose to take actions in the dilemmas.

Based on the time course of neural activity elicited by moral judgments for different agents when the agent chose to take the action or take no action during the dilemma, the present study found that four ERP components, including N1, P2, N2, and the LPC, were associated with the moral judgment. There was no difference observed for the N1, P2, or N2 amplitudes. The divergence of waveforms elicited by the four experimental conditions was most clearly pronounced on LPC. According to dual-process theory (Greene et al., 2001, 2004; Haidt, 2001, 2007), moral judgment is considered as a multi-level process involving the automatic emotional process supporting moral intuition, and the controlled cognitive process supporting moral reasoning. Previous ERP studies concerning moral processing proposed that early components were related to the fast and automatic process of moral intuition or the emotional responses triggered by moral stimuli (Sarlo et al., 2012; Lahat et al., 2013; Yoder and Decety, 2014; Cowell and Decety, 2015; Gui et al., 2016; Pletti et al., 2019). For example, Yoder and Decety (2014) observed that compared with antisocial actions, moral judgments for prosocial actions elicited larger N1 and N2, which were proposed to associate with emotional salience.

In the present study, no significance difference was observed in the early components. It suggested that there might be an identical early emotional processing in the moral judgments under the four current experimental conditions. The results were inconsistent with previous studies concerning moral processing, in which the divergences of ERP waveforms were observed on early components (Sarlo et al., 2012; Lahat et al., 2013; Yoder and Decety, 2014; Cowell and Decety, 2015; Gui et al., 2016; Pletti et al., 2019). These divergent results might be attributed to the type of the tasks used in different studies. Previous studies focused on the comparisons between the moral processing for prosocial actions and antisocial actions, or between the instrumental dilemmas and incidental dilemmas. The emotional response elicited by the tasks could be distinct between these two cases. As Greene et al. (2001) proposed, emotional response could be the crucial difference between the trolley and footbridge dilemmas. For instance, pushing an innocent person off the bridge in the footbridge dilemma was more emotionally salient than hitting a switch that would cause a trolley to run over an innocent person in the trolley dilemma. The sort of violation in the footbridge dilemma was more personal and emotional than the other type of dilemma, which also led to increased RTs (Greene et al., 2001). In the present study, we used 30 moral dilemmas modified from Lotto et al. (2013). Although the types of agents and behaviors were different among the four experimental conditions, all the 30 dilemmas required participants to make moral judgments in scenarios similar to the footbridge dilemma. That is, the only way to save some people in danger was killing or harming an innocent victim personally and directly. Thus, we proposed that, in the present study, the emotional responses in the early stage elicited by moral judgments under the four experimental conditions were similar. It could also account for the patterns in behavioral results: there was no significant difference in RTs among the four experimental conditions. These results suggested that the interaction between ToM and moral judgment did not influence the early stages during moral judgment processing.

Moving to the later time window, the results revealed a significant main effect of behavior types for the LPC amplitude. Specifically, the moral judgment for the agent who chose to take the action of killing or harming an innocent person but saving more people elicited a larger LPC than the moral judgment for the agent who chose to take no action but let some people die. It is acknowledged that the P3-related late neural activity usually represents higher-level cognitive processes such as cognitive reappraisal, cognitive control, or saliency processing (Polich, 2007; Amodio et al., 2014; Cowell and Decety, 2015). Based on the later time window and the frontal scalp distribution, the LPC in the present study could also be a member of the P3 family. Previous ERP studies indicated that the late positive potential (P3 or LPP) was related to moral judgments. Yoder and Decety (2014) reported that the moral judgment for prosocial actions elicited a greater frontal LPP (300–600 ms) than antisocial actions. The authors argued that the LPP reflected the cognitive re-appraisal in moral evaluation. In Gui et al.’s (2016) study, participants were asked to make moral judgments of people’s behavior as presented in pictures. The results found that the early LPP (350–420 ms) was strongly influenced by emotional arousal, whilst the later slow wave (450–650 ms) was influenced by emotional arousal and moral content. In general, the LPP reflects the allocation of cognitive resources and appraisal of motivationally salient stimuli (for reviews see Hajcak et al., 2010). Considering the long duration and the late time window, we proposed that the LPC (500–900 ms) in the present study reflected the late controlled cognitive process in the dual-process theory of moral judgment (Greene et al., 2001, 2004). The greater LPC elicited by the moral judgments for the action conditions might indicated that the corresponding cognitive appraisal was being allocated more resources than that for the inaction conditions. This argument was consistent with neuroimaging evidence reporting that the utilitarian choice induced greater activation in associative brain areas than the non-utilitarian choice (Greene et al., 2004), and supported by the DDA in moral processing: if an action caused predictable harm, it was judged worse than an omission (Cushman et al., 2006; Woollard, 2013).

In the present study, we investigated the interaction between ToM and moral judgment by comparing the moral judgments for self and for others in dilemmas. The most noteworthy finding was the significant interaction between agent types and behavior types on the frontal LPC. Results revealed that the moral judgment for others elicited a larger LPC than that for self when the agents took actions of killing or harming an innocent person to save more people, whilst the difference was not significant when the agents took no action. Since the influence of ToM was operated by contrasting the agent types during moral dilemmas, the LPC divergence between moral judgments for self and for others might index the different ToM processes involved in moral judgment. The results suggested that the effect of ToM arose at the later stage during moral judgment processing. It was consistent with previous ERP studies comparing the moral decision-making for people with different interpersonal closeness levels. The divergences of P3 or LPP were observed between moral decision-making for different agents (Chen et al., 2009; Zhan et al., 2018). The larger amplitudes of P3 or LPP were believed to index the greater attentional resources and cognitive effort required to solve conflicts involved in moral processing. Although the polarity of the LPC effect in the present study was not the same with Chen et al.’s study (2009), it might be attributed to the paradigm diversity and the different electrodes chosen for statistical analysis. Intriguingly, the frontal LPC divergence was reported in our previous studies contrasting the ToM reasoning for self and for others, which was assumed to reflect the decoupling mechanism to distinguish the self from the others in ToM processes (Jiang et al., 2016, 2021). Thus, the dissociation of LPC occurring in moral judgment for self and for others possibly indicated that participants allocated more cognitive resources to the ToM processing of understanding the other person’s mental states. Specifically, the dissociation was only observed when the agent chose to take actions since more cognitive resources were required to resolve the stronger conflict induced by the utilitarian choice in action conditions rather than the non-utilitarian choice in inaction conditions (Greene et al., 2004; Sarlo et al., 2012, 2014).

This assumption was confirmed by the negative correlation between the RMET scores and the LPC amplitudes observed in the current study. The results suggested that the larger the LPC amplitudes were, the lower scores were obtained by participants in the RMET. It indicated that the individuals with the poorer mentalizing ability had to allocate more cognitive resources to understand other people’s feelings in the moral dilemma. This finding was also consistent with previous studies revealing that the individual differences in dispositional empathy or cognitive empathy were correlated with LPP amplitudes (Cheng et al., 2014; Yoder and Decety, 2014; Decety et al., 2015). Importantly, individual differences in mentalizing ability were correlated with the amplitudes of the late component but not the early component of neural responses to the moral judgment. Based on the dual-process theory of moral judgment (Greene et al., 2001, 2004), these findings might demonstrate that the ToM affected the late controlled cognitive process during moral judgments, rather than the early automatic process. The patterns of the interaction between moral judgment and ToM were also in line with the previous ERP studies reporting that the later-stage component such as LPP was affected by perspective-taking or empathy whilst the early-stage component was not (Li and Han, 2010; Decety et al., 2015).

There are several limitations in the present study that need to be acknowledged. First, the small sample size could not provide sufficient power for the correlational results. Second, although the task of RMET is a widely used tool measuring ToM, it mainly focuses on the understanding of other people’s feelings. Since ToM is a multi-dimensional conception including desire, belief, intention and feeling, future research should use other tasks that assess different dimensions of ToM to obtain a more integrated interpretation of the interaction between ToM and moral judgment.

## Conclusion

By contrasting the electrical activity elicited by moral judgments for self and for others when the agent chose to take the action or take no action during the dilemma, the present study provides important insights into the temporal neural dynamics of the interaction between moral judgment and ToM. Results found that during the 500–900 ms time period, the frontal LPC elicited by moral judgment for others was more positive than for self when the agents chose to take actions of killing or harming an innocent person but saving more people in moral dilemmas. It was assumed to represent the extra cognitive resources allocated to the ToM processing in moral judgments for others. There was no difference observed on the early components of N1, P2, and N2. These findings suggested that the ToM modulated the late controlled process but not the early automatic process during moral judgments.

## Data Availability Statement

The raw data supporting the conclusions of this article will be made available by the authors, without undue reservation.

## Ethics Statement

The studies involving human participants were reviewed and approved by the Ethics Committee of the School of Marxism at Guangxi University. The patients/participants provided their written informed consent to participate in this study.

## Author Contributions

QJ: study design, data collection, data analyzing, and manuscript writing. LZ: data collection and data analyzing. QW: theoretical discussion and manuscript writing. WL: data analyzing. All authors contributed to the article and approved the submitted version.

## Conflict of Interest

The authors declare that the research was conducted in the absence of any commercial or financial relationships that could be construed as a potential conflict of interest.

## Publisher’s Note

All claims expressed in this article are solely those of the authors and do not necessarily represent those of their affiliated organizations, or those of the publisher, the editors and the reviewers. Any product that may be evaluated in this article, or claim that may be made by its manufacturer, is not guaranteed or endorsed by the publisher.
